# The effects of *Cissus quadrangularis* on bone-related biomarkers in humans: a systematic review and meta-analysis

**DOI:** 10.1186/s12906-025-04995-8

**Published:** 2025-07-24

**Authors:** Mingkwan Na Takuathung, Jakkrit Aisara, Suphunwadee Sawong, Nut Koonrungsesomboon

**Affiliations:** 1 Department of Pharmacology, Faculty of Medicine, Chiang Mai University, Chiang Mai, 50200 Thailand; 2 Clinical Research Center for Food and Herbal Product Trials and Development (CR-FAH), Faculty of Medicine, Chiang Mai University, Chiang Mai, 50200 Thailand

**Keywords:** *Cissus quadrangularis*, Bone, Bone markers, Systematic review, Meta-Analysis

## Abstract

**Background:**

Maintaining bone health is vital, particularly for aging populations prone to bone disorders. *Cissus quadrangularis* (CQ), a traditional medicinal plant, is increasingly recognized for its potential benefits in promoting bone health, including the effects on bone-related biomarkers promoting bone healing and bone density improvement. This study aimed to evaluate the effects of CQ on bone-related biomarkers in humans as the primary objective and to assess its impact on other clinical outcomes as a secondary objective.

**Methods:**

A systematic search of PubMed, Embase, Scopus, and the Cochrane Library databases was conducted up to April 2025. This systematic review included studies with participants receiving *Cissus quadrangularis*, those reporting bone biomarkers, and those with a randomized controlled design involving human participants. No restrictions were placed on age, sex, disease severity, or administration route. The quality of the studies included was assessed using the revised Cochrane risk-of-bias tool (RoB-2), while the overall strength of evidence was appraised using the GRADE approach. The summary effect measure was the standardized mean difference (SMD) with 95% confidence intervals (95% CI).

**Results:**

Seven studies, comprising a total of 354 participants, met the inclusion criteria for meta-analysis. The analysis revealed that CQ intervention significantly elevated serum parathyroid hormone levels (SMD = 1.23; 95% CI = 0.79 to 1.67; *p* < 0.0001). However, the CQ intervention did not result in significant changes in other bone-related biomarkers, including calcium, phosphorus, and serum alkaline phosphatase. The RoB-2 revealed that most studies had some concerns about bias, with two evaluated as having a high risk. The certainty of evidence was very low for all four parameters assessed by GRADE.

**Conclusion:**

The CQ intervention was significantly associated with increased serum parathyroid hormone levels, with no significant effects on other bone-related biomarkers. Although there were a limited number of studies, these findings suggest its potential for modulating bone health; however, further research is needed to confirm these results and explore its clinical applications.

**Trial registration:**

The study protocol was prospectively registered with PROSPERO, and the registration number is CRD42023435351.

**Clinical trial number:**

Not applicable.

**Supplementary Information:**

The online version contains supplementary material available at 10.1186/s12906-025-04995-8.

## Introduction

Bone remodeling is a dynamic physiological process essential for maintaining skeletal integrity, repairing microdamage, and regulating calcium homeostasis [1]. This continuous cycle involves the coordinated actions of osteoclasts, which remove old bone, and osteoblasts, which form a new bone matrix through three sequential phases: resorption, reversal, and formation [2]. Complex interactions among several key biochemical markers govern the regulation of bone metabolism. Parathyroid hormone plays a central role in calcium homeostasis, working in concert with serum alkaline phosphatase and phosphorus to maintain optimal bone mineralization [2]. Disruptions in this balance can lead to bone health disorders that affect diverse populations due to various factors, including metabolic disorders, nutritional deficiencies, physical inactivity, certain medications, and specific medical conditions [3]. In 2020, the global prevalence of osteoporosis was reported to be 18.3% (95% CI 16.2–20.7%), highlighting the necessity of seeking treatment for individuals with osteoporosis and implementing measures to prevent fractures [4].

*Cissus quadrangularis* L. (CQ), regionally known as Phet Sang Khat in Thailand, Hadjod in India, and Veldt grape, has been traditionally valued in Ayurvedic medicine for its role in promoting bone healing [5, 6]. Several clinical studies suggest that CQ extracts, when administered over several weeks, may accelerate bone fracture healing [6–8]. These findings are further supported by various in vitro and animal studies exploring its mechanisms and effects on bone health [5, 6, 9] highlighting CQ’s potential as an alternative therapy for bone-related conditions. CQ’s bioactive compounds, including alkaloids, flavonoids, and triterpenoids, are thought to enhance osteogenesis, reduce bone resorption, and modulate key bone-related biomarkers [10].

A previous systematic review and meta-analysis evaluated the efficacy and safety of CQ across various conditions [8]. The study included 1,108 patients and investigated both CQ alone and in combination products compared with placebo or other treatments. Outcomes included symptoms of hemorrhoids, bone pain, body weight, lipid profiles, and fasting blood sugar. The results indicated significant improvements in bone pain and metabolic parameters with CQ combinations. Although significant effects were obtained, the pooled results for each outcome were based on studies using different formulations of CQ products, comparators, and a relatively small sample size, which may have contributed to imprecise estimates or findings influenced by variability and uncertainty. Despite its promising therapeutic potential, significant gaps remain in understanding its effects on bone health at the molecular and systemic levels.

The present study primarily aimed to address existing knowledge gaps by conducting a systematic review and meta-analysis to evaluate the effects of CQ on bone-related biomarkers across diverse populations and bone conditions. Additionally, clinical outcomes were examined as a secondary objective. The findings are expected to inform future research and support the development of herbal medicinal products in this field.

## Materials and methods

### Study protocol, registration, and ethical approval

This study was conducted in line with the Cochrane Handbook for Systematic Reviews of Interventions [11] and reported following the Preferred Reporting Items for Systematic Reviews and Meta-Analyses (PRISMA) guideline [12]. The study protocol was prospectively registered with the PROSPERO (CRD42023435351). The Research Ethics Committee of the Faculty of Medicine, Chiang Mai University granted an exempt research determination to this study protocol (EXEMPTION-0214/2022).

### Search strategy and eligibility criteria

The data collection process involved comprehensive searches across several databases, including PubMed, Embase, Scopus, and the Cochrane Library (with the last search update on April 12, 2025). These databases were thoroughly queried using a tailored set of search terms to capture the relevant literature. The terms focused on *Cissus quadrangularis* (CQ) in relation to various bone diseases, utilizing combinations as follows: “(Cissus OR quadrangularis OR *Cissus quadrangularis* OR Asthisrankhala OR Pet-Sang-Kart) AND (Bone OR Bone disease OR Bone fractures OR Osteoporosis OR Osteopenia OR Osteolysis OR Bone density OR Bone resorption OR Bone loss OR Bone remodeling OR Bone turnover OR Bone regeneration OR Bone mineral OR Bone mineral density OR BMD OR Bone mineral content OR BMC)”. These search terms were consistently applied across four databases to comprehensively capture relevant literature, with no language restrictions imposed.

The inclusion criteria were: (1) studies in which participants received *Cissus quadrangularis*; (2) studies reporting bone-related biomarkers; and (3) studies conducted exclusively on human participants using a randomized controlled design. Studies were selected without restrictions on participants’ age, sex, disease severity, or route of administration. Exclusion criteria included review articles, case reports, expert opinions, conference proceedings, and book chapters.

### Data extraction

Titles and abstracts of studies, sourced through a systematic search strategy and additional sources, were independently screened by review authors (JA and SS) to identify studies that potentially met the inclusion criteria. The full texts of these eligible studies were then retrieved and independently assessed by the review authors (JA and SS) for eligibility. Any disagreements regarding study eligibility were constructively resolved through discussions (with MN and NK).

For each selected study, data extraction included several key parameters: (1) study information (e.g., first author’s name, publication year, and trial registration); (2) study design and setting (e.g., design, country of origin, sample size, and length of follow-up); (3) population characteristics (e.g., disease/condition, age, and sex); (4) characteristics of the intervention and its comparator (e.g., route of administration, dose, frequency, and duration); and (5) outcomes of interest. The outcomes of interest included serum osteogenic biomarkers (e.g., serum parathyroid hormone, serum calcium, serum phosphorus, and serum alkaline phosphatase) and any other biomarkers specific to bone diseases/conditions. Parameters related to the clinical presentation of bone disorders were included in exploratory analyses. When data in an original publication were missing or unclear, attempts were made to contact the corresponding author for clarification.

### Risk of bias assessment

Potential bias in randomized trials was assessed using the revised Cochrane risk-of-bias tool for randomized trials (RoB-2) [13]. This tool enabled a thorough appraisal of five key areas: (1) bias arising from the randomization process; (2) bias related to deviations from intended interventions; (3) bias due to missing outcome data; (4) bias in outcome measurement; and (5) bias in the selection of reported results. Bias levels were categorized as ‘low,’ ‘some concerns,’ or ‘high.’ Any disagreements between review authors regarding bias assessments were resolved through discussion, and if consensus could not be reached, a third review author was consulted for a final decision.

### Statistical analyses

Statistical analysis was conducted using R, version 2023.09.0 + 463 (RStudio). Continuous data were presented as standardized mean differences (SMD) with 95% confidence intervals (CI). Missing data, including the standard deviation (SD) of some outcomes, were imputed following the method for imputing standard deviations for changes from baseline. The correlation coefficient (Corr_E_) was calculated using the outcome means and SDs from other related studies, if applicable. When no related study data were available, a Corr_E_ of 0.5 was used, as recommended by the Cochrane guidelines [14].

Small study effects, including publication bias, were not assessed since the number of studies is less than 10. Additionally, subgroup analysis was not performed since there were no substantial heterogeneities, as assessed by I² values (with I² > 40% considered substantial), Cochran’s Q tests (with *p* < 0.05 considered significant), and between-study variance (τ²).

Sensitivity analyses were conducted using leave-one-out analysis and comparing between fixed effect and random effect analysis methods. The leave-one-out analysis was performed based on the number of included studies and the degree of clinical and methodological homogeneity. We considered a minimum of 3 studies appropriate appropriate for sensitivity analysis when the elements, including population, intervention, and comparator, were highly comparable, which enhances the validity and interpretability of the pooled results.

### GRADE assessment for developing recommendations

In this study, we applied the GRADE (Grading of Recommendations, Assessment, Development, and Evaluation) approach to assess the quality of the evidence for clinical practice recommendations [15]. In brief, evidence from randomized controlled trials initially rated as high quality was downgraded based on the risk of bias, indirectness, imprecision, inconsistency (heterogeneity), or publication bias.

## Results

**Fig. 1:**
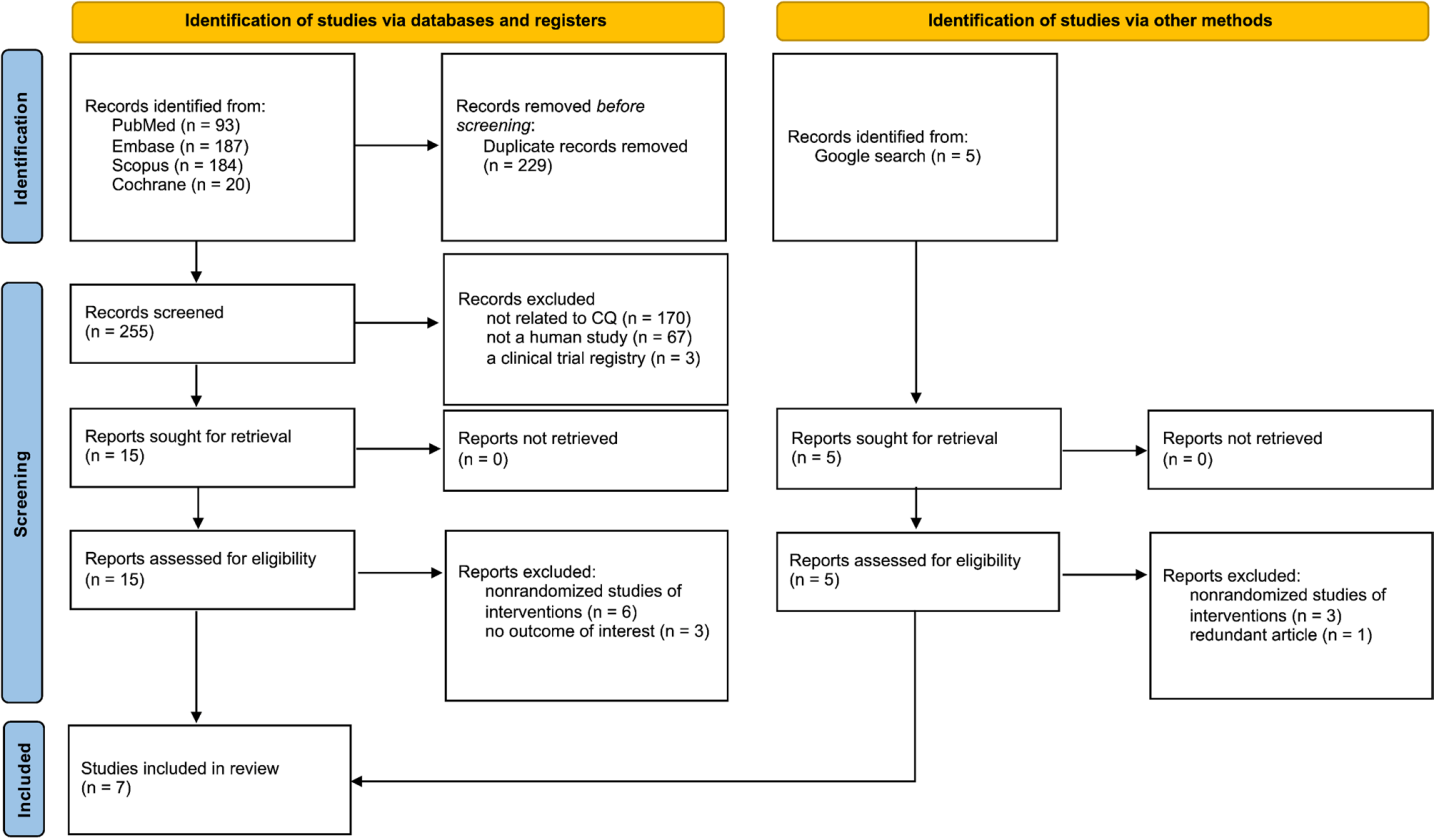
PRISMA flow diagram of the study selection process

The PRISMA flow diagram presents the study selection process for this systematic review and meta-analysis (Fig. 1). Initially, 484 records were identified from the database, along with six additional records from other sources (i.e., websites). After removing duplicates, 255 articles were screened based on titles and abstracts, and 15 studies were selected for full-text review and eligibility assessment. Finally, seven studies involving 354 participants were included in the analysis.

The general characteristics of the studies included in the meta-analysis are summarized in Table 1. The participant ages ranged from 12 to 70 years, with sample sizes varying from 3 to 46 participants. The population across the studies had various bone conditions, including bone fractures (*n* = 4), oral cavity bone diseases (*n* = 2) (i.e., mandibular alveolar ridge distraction (*n* = 1), implant placement (*n* = 1)), and metabolic bone disease (*n* = 1) (i.e., osteopenia). Geographically, most studies were primarily conducted in Asia (*n* = 6), particularly in India (*n* = 5). The CQ intervention was predominantly administered by oral route in different dosages, ranging from 500 mg/day to 10 g/day, with intervention durations spanning from 4 to 24 weeks. Follow-up periods ranged from 31 days to 24 weeks. Outcome measures included serum calcium (Ca), phosphorus (P), alkaline phosphatase (ALP), parathyroid hormone (PTH), as well as C-terminal telopeptide (CTX), procollagen type 1 N-terminal propeptide (P1NP), and osteopontin. The control groups included a placebo (an inactive substance) for 4 studies and amoxicillin/clavulanic acid (antibiotics) for 1 study. There were two studies that compared CQ with no intervention to assess baseline effects. According to the RoB-2, most studies raised some concerns regarding bias, with two studies identified as having a high risk of bias (Figure S1).

**Fig. 2:**
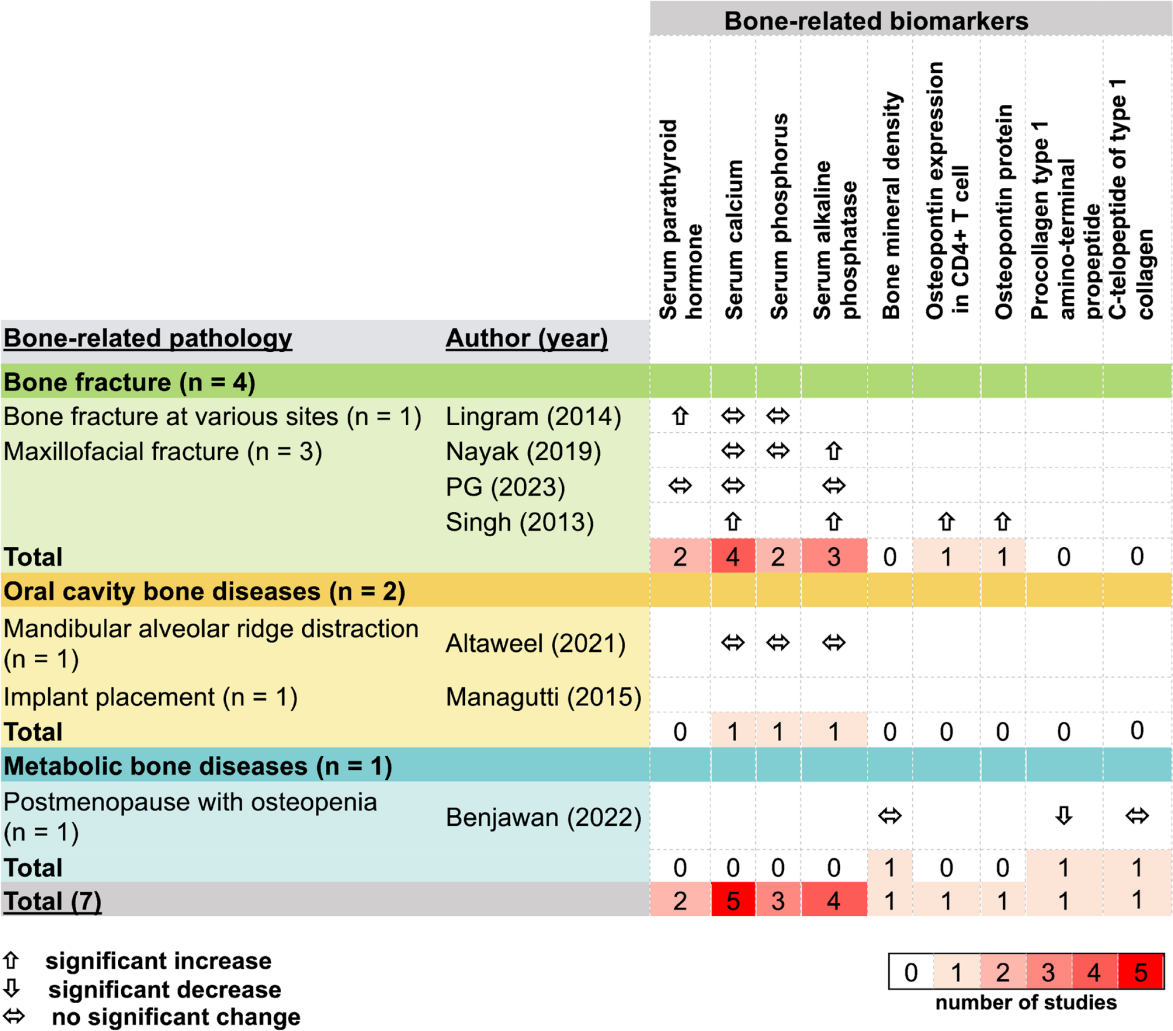
Overview of bone outcome parameters across the included studies

The qualitative synthesis identified multiple parameters measured across 7 studies, involving three major population groups: those with bone fractures (*n* = 4), oral cavity bone diseases (*n* = 2), and metabolic bone diseases (*n* = 1). The majority of measured parameters included serum calcium (*n* = 5), serum alkaline phosphatase (*n* = 4), serum phosphorus (*n* = 3), and serum parathyroid hormone (*n* = 2) (Fig. 2). Parameters related to the clinical presentation of bone disorders are summarized in Figure S2. Clinical outcomes were reported across studies involving bone fractures, oral cavity bone diseases, and metabolic bone disease (Figure S2). Overall, pain was the most frequently reported clinical outcome (5 studies), while bone height, defect resolution, and bone mineral density were each reported by one study.

**Fig. 3:**
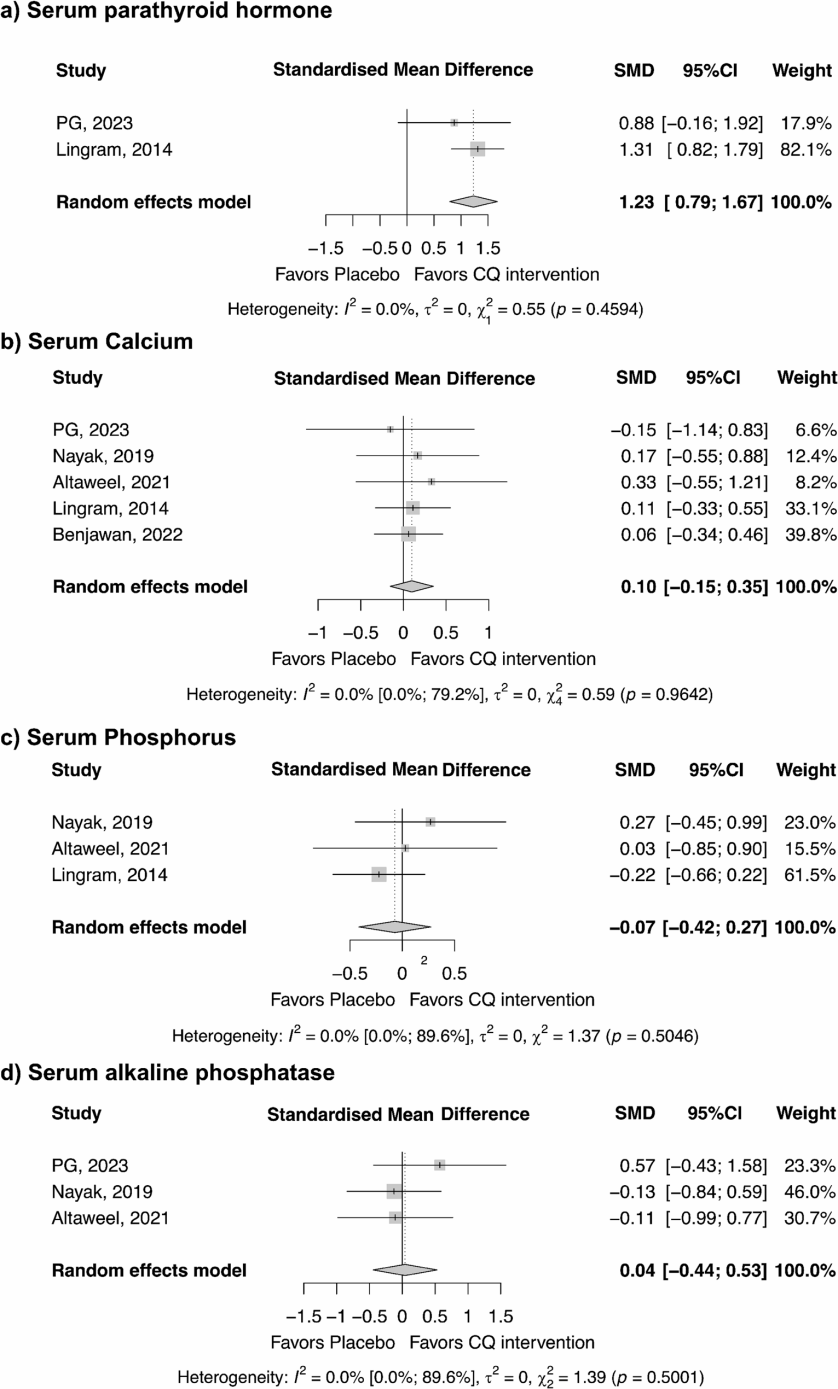
Meta-analysis of *Cissus quadrangularis* interventions on bone-related biomarkers: (**a**) serum parathyroid hormone, (**b**) serum calcium, (**c**) serum phosphorus, and (**d**) serum alkaline phosphatase

The meta-analysis evaluated the effects of CQ on four bone-related biomarkers: serum parathyroid hormone, serum calcium, serum phosphorus, and serum alkaline phosphatase. The analysis revealed a significant increase in serum parathyroid hormone levels following the CQ intervention when compared to the control (SMD = 1.23; 95% CI = 0.79 to 1.67; *p* < 0.0001). However, no significant differences were observed between the CQ intervention and the control for the other parameters (Fig. 3). Sensitivity analyses confirmed the robustness of the results, as no significant change was observed after performing leave-one-out analysis and comparing fixed-effect and random-effects models (Figure S3-4). The GRADE assessment of evidence certainty is summarized in Table 2, with all four parameters rated as having very low certainty. There was no statistical heterogeneity among the included studies, as indicated by an I² value of 0%, τ² of 0, and non-significant Chi² test results (p-values ranging from 0.4594 to 0.9642).

## Discussion

The current systematic review and meta-analysis provides a comprehensive synthesis of clinical evidence of CQ on bone-related biomarkers across diverse populations and bone conditions. The analysis highlights that CQ interventions have been predominantly studied in three pathologic conditions: bone fractures, oral cavity bone diseases, and metabolic bone conditions.

Bone fractures and oral cavity bone diseases usually involve bone healing and regeneration. These conditions share a common pathway of acute bone injury or surgical manipulation, triggering biological processes such as inflammation, callus formation, and bone remodeling [16, 17]. To monitor these processes, biomarkers of bone homeostasis, including serum parathyroid hormone (PTH), calcium (Ca), phosphorus (P), and alkaline phosphatase (ALP), are commonly used due to their critical roles in regulating mineral metabolism and bone turnover [18].

In the context of CQ supplementation, the selection of biomarkers such as PTH, Ca, P, and ALP is crucial for evaluating CQ’s potential effects on bone health and endocrine regulation, particularly given its traditional use in supporting bone health and aiding fracture healing [19]. PTH is a key regulator of calcium homeostasis and bone remodeling. Since CQ has osteogenic effects, it may influence PTH levels, making its monitoring important during the healing process [20]. Calcium, a major structural component of bone, is essential for mineralization, and fluctuations in its serum levels can indicate disturbances in bone metabolism. Monitoring calcium is therefore important for evaluating CQ’s potential to support bone formation [21]. Phosphorus, which is vital for bone mineralization and hydroxyapatite formation, may be influenced by CQ’s effects on bone metabolism, making it essential to monitor phosphorus levels during the regeneration phase [22]. Lastly, ALP, a marker of osteoblast activity and bone formation [18, 23] provides insight into the extent of bone formation stimulated by CQ supplementation, given its role in promoting osteogenesis [24].

In contrast, metabolic bone disease, specifically osteopenia, involves chronic systemic bone loss primarily due to hormonal imbalances, such as estrogen deficiency in postmenopausal women [25]. This condition is characterized by increased bone resorption relative to bone formation and is evaluated using bone turnover markers such as C-terminal telopeptide (CTX) and procollagen type 1 N-terminal propeptide (P1NP) [26]. However, since CQ is primarily associated with improving acute bone healing and regeneration, our study focuses on biomarkers (PTH, Ca, P, and ALP) that are directly relevant to these regenerative processes, rather than bone turnover markers primarily linked to chronic systemic bone loss.

Although the number of studies was limited, with only seven studies covering four main biomarkers, the results indicated that CQ administration was associated with a significant increase in serum parathyroid hormone levels, suggesting a potential role in modulating bone metabolism. These findings underscore the need for further well-designed studies to confirm CQ’s effects and clarify its therapeutic potential in bone-related conditions.

PTH is a central regulator of calcium homeostasis and bone metabolism [27], exerting its effects by promoting calcium reabsorption in the kidneys, stimulating vitamin D activation to enhance intestinal calcium absorption, and mobilizing calcium from bones when systemic levels are low [28]. In our meta-analysis, CQ administration was consistently associated with elevated PTH levels, yet this increase occurred without significant changes in serum calcium, phosphorus, or ALP compared to controls. This dissociation points to a mechanism of PTH regulation that operates independently of classical calcium feedback, possibly involving altered sensitivity of the parathyroid gland through modulation of the calcium-sensing receptor (CaSR) [29]. Such a pattern is reminiscent of early-stage primary hyperparathyroidism, where PTH elevation can precede detectable changes in serum calcium and is associated with increased risk for bone remodeling abnormalities and bone loss [30].

Additionally, the impact of PTH on bone is highly dependent on the pattern and duration of hormone exposure. Intermittent elevations of PTH, as achieved in certain therapeutic regimens, stimulate osteoblast activity and bone formation, whereas sustained high levels promote bone resorption and loss of bone density [31]. Our findings, together with preclinical evidence, suggest that CQ may enhance bone formation by promoting PTH pulsatility and upregulating key pathways such as insulin-like growth factor 1 (IGF-1) [32]. IGF-1 is a critical mediator of bone remodeling, supporting osteoblast proliferation, differentiation, and survival, and its expression is influenced by PTH signaling [32]. Supporting this, previous in vitro studies have shown that the ethanolic extract of CQ positively regulates components of the IGF system in human osteoblast-like SaOS-2 cells [33]. Additionally, a 300 µg/mL petroleum ether extract of CQ was found to enhance the proliferation rate of marrow mesenchymal stem cells and promote osteoblastogenesis [34].

Clinically, these findings have important implications. Chronic elevation of PTH, even in the absence of hypercalcemia, may increase the risk of cortical bone loss and osteoporosis, as observed in normocalcemic primary hyperparathyroidism [24, 35]. Conversely, intermittent rises in PTH could benefit trabecular bone, similar to the effects seen with teriparatide therapy, which improves trabecular microarchitecture but may differentially affect cortical bone [36]. Nevertheless, caution is warranted, as prolonged or excessive elevation of parathyroid hormone could shift the balance toward bone resorption, potentially compromising bone density [37]. Our observation highlights the need for further research to elucidate the mechanisms through which CQ modulates PTH and to better understand its broader implications for bone health and systemic physiology across diverse patient populations.

The results of this study should be interpreted in light of its limitations. First, the relevant studies were identified through a systematic search of four databases. Consequently, it is possible that articles available exclusively in other databases were not included in our analysis. Expanding database searches, such as clinical trial registry platforms, and including grey literature and conference papers, will improve the comprehensiveness of the study pool. Second, the sensitivity analysis could not be performed since the limited number of studies made it difficult to draw strong conclusions regarding the effects of CQ on bone-related biomarkers, particularly across the diverse pathological conditions examined. The wide variability in these conditions adds further complexity, limiting the generalizability of CQ’s impact. Third, inconsistencies in the extraction methods used for CQ across the studies, along with the absence of clearly reported extraction methods, hindered comparisons and the evaluation of the effects of different extraction techniques or the determination of an optimal CQ dosage for bone health benefits. This variability restricts insights into which specific extraction methods or dosages are most effective. Future RCT studies should standardize data extraction methods in order to perform subgroup analyses, which will allow for more meaningful comparisons and greater precision in findings. Fourth, the risk of bias assessment using the RoB 2 tool revealed that several studies had some concerns, particularly in domain 1 (randomization process) and domain 2 (deviations from intended interventions). These issues were noticed as a result of insufficient information about allocation concealment and lack of blinding, which could introduce performance or selection bias. The GRADE approach rated the certainty of evidence as very low, primarily due to concerns about the risk of bias and imprecision, thereby limiting confidence in the findings and their interpretation. Lastly, we were unable to conduct formal statistical tests (e.g., Egger’s test [38]) to assess the presence of small-study effects or publication bias. This limitation arose from the relatively small number of datasets included in the meta-analysis, as Egger’s test generally requires at least 10 studies for robust analysis [39, 40]. Additionally, substantial heterogeneity among the included studies and the limited availability of primary research data necessitate cautious interpretation of the results.

## Conclusions

In summary, this systematic review and meta-analysis presented promising findings regarding the potential impact of CQ extract in promoting bone health and modulating certain bone conditions. Overall, CQ appears to increase serum parathyroid hormone levels. However, further research is warranted to validate these findings and to explore the underlying mechanisms involved.

**Table 1:** General characteristics of the included studies

Study	Context		Participant				Intervention feature				Outcome measure
	Design	Study location	Condition/ disease	Participant’s age	sample size of intervention (male)	sample size of control (male)	Intervention	Control	Route of administration (frequency)	Duration (follow up)	Outcome parameter
Altaweel (2021)	RCT (triple-blinded)	Egypt	Mandibular alveolar ridge distraction	51–58 years	10 (6)	10 (5)	CQ 500 mg/day	Placebo	Oral (o.d.)	6 weeks (6 months)	Serum calcium, serum phosphorus, and serum alkaline phosphatase
Benjawan (2022)	RCT (single-blinded)	Thailand	Postmenopausal women with osteopenia	51–60 years	44/44	46	1. CQ 1.2 g/day 2. CQ 1.6 g/day	Placebo	Oral (b.i.d.)	24 weeks (24 weeks)	Serum Calcium, CTX and P1NP
Lingram (2014)	RCT (open-label)	India	Bone fracture (various sites)	12–70 years	40 (23)	40 (23)	CQ 10 g/day	Placebo	Oral (t.i.d.)	30 days (31 days)	Serum calcium, serum phosphorus, and serum parathyroid hormone
Managutti (2015)	RCT (open-label)	India	Implant placement	18–55 years	3 (3)	3 (3)	CQ 500 mg/day	Amoxicillin 500 mg and clavulanic acid 125 mg, and diclofenac sodium	Oral (b.i.d.)	8 weeks (8 weeks)	Serum alkaline phosphatase
Nayak (2019)	RCT (open-label)	India	Mandibular fracture	20–40 years	15 (13)	15 (13)	CQ 1000 mg/day	None	Oral (b.i.d.)	6 weeks (6 weeks)	Serum calcium, serum phosphorus, and serum alkaline phosphatase
PG (2023)	RCT (open label)	India	Maxillofacial fracture	18–60 years	8 (8) / 8(7)	8 (8)	1. CQ 1000 mg/day and DS 800 mg/day 2. Teriparatide 20 ug/day (subcutaneous)	None	Oral (b.i.d) and subcutaneous (o.d.)	4 weeks (12 weeks)	Serum calcium, serum alkaline phosphatase, and serum parathyroid hormone
Singh (2013)	RCT (double-blinded)	India	Mandibular fracture	N/A	30 (NR)	30 (NR)	CQ 1200 mg/day	Placebo	Oral (b.i.d.)	8 weeks (3 months)	Serum calcium, serum alkaline phosphatase, osteopontin expression in CD4 + T cell, and osteopotin protrein

CD4+, cluster of differentiation 4; CQ, Cissus quadrangularis; CTX, C-telopeptide of type 1 collagen; DS, Dalbergia sissoo; NR, not reported; P1NP, procollagen type 1 amino-terminal propeptide; RCT, randomized controlled trial

**Table 2:** GRADE assessment of the certainty of evidence in this systematic review and meta-analysis

Certainty assessment							No. of patients		Effect			
**No. of studies**	**Study design**	**Risk of bias**	**Inconsistency**	**Indirectness**	**Imprecision**	**Other considerations**	**CQ**	**Placebo**	**Relative** **(95% CI)**	**Absolute** **(95% CI)**	Certainty	Importance
**Serum parathyroid hormone**												
2	randomized trials	serious	not serious	serious	very serious	publication bias strongly suspected	48	48	-	SMD **1.23 SD higher** (0.79 higher to 1.67 higher)	⨁◯◯◯ Very low	NOT IMPORTANT
**Serum calcium**												
5	randomized trials	serious	not serious	serious	very serious	publication bias strongly suspected	145	109	-	SMD **0.1 SD higher** (0.15 lower to 0.35 higher)	⨁◯◯◯ Very low	NOT IMPORTANT
**Serum phosphorus**												
3	randomized trials	serious	not serious	serious	very serious	publication bias strongly suspected	65	65	-	SMD **0.07 SD lower** (0.42 lower to 0.27 higher)	⨁◯◯◯ Very low	NOT IMPORTANT
**Serum alkaline phosphatase**												
3	randomized trials	serious	not serious	serious	very serious	publication bias strongly suspected	33	33	-	SMD **0.04 SD higher** (0.44 lower to 0.53 higher)	⨁◯◯◯ Very low	NOT IMPORTANT

## Electronic supplementary material

Below is the link to the electronic supplementary material.

Supplementary Material 1

Supplementary Material 2

Supplementary Material 3

## Data Availability

The datasets used and/or analyzed during the current study are available from the corresponding author upon reasonable request.
