# Supplementary material for: The effects of Cissus quadrangularis on bone-related biomarkers in humans: a systematic review and meta-analysis
Source: BMC Complement Med Ther. 2025 Jul 24;25:286. doi: 10.1186/s12906-025-04995-8 (PMC12288206; doi:10.1186/s12906-025-04995-8)
Supplement: Supplementary file 1 — Supplementary Material 1 [file 12906_2025_4995_MOESM1_ESM.pdf]

Supplementary material 1

Study ID	Outcome	D1	D2	D3	D4	D5	Overall
Altaweel, 2021	Serum alkaline phosphatase	+	+	+	+	+	+
Altaweel, 2021	Serum calcium	+	+	+	+	+	+
Altaweel, 2021	Serum Phosphorus	+	+	+	+	+	+
Benjawan, 2022	C-telopeptide of type 1 collagen	+	!	+	+	+	!
Benjawan, 2022	Procollagen type 1 amino-terminal propeptide	+	!	+	+	+	!
Benjawan, 2022	Serum calcium	+	+	+	+	+	+
Lingram, 2014	Serum calcium	!	!	+	+	+	!
Lingram, 2014	Serum phosphorus	!	!	+	+	+	!
Lingram, 2014	Serum parathyroid hormone	!	!	+	+	+	!
Managutti, 2015	Serum alkaline phosphatase	!	!	+	+	!	-
Nayak, 2019	Serum alkaline phosphatase	!	!	+	+	+	!
Nayak, 2019	Serum calcium	!	!	+	+	+	!
Nayak, 2019	Serum phosphorus	!	!	+	+	+	!
PG, 2023	Serum alkaline phosphatase	+	+	+	+	+	+
PG, 2023	Serum calcium	+	+	+	+	+	+
PG, 2023	Serum parathyroid hormone	!	+	+	+	+	!
Singh, 2013	Osteopontin expression in CD4+ T cell	+	-	-	!	!	-
Singh, 2013	Osteopotin protrein	+	-	-	!	!	-
Singh, 2013	Serum alkaline phosphatase	+	-	-	!	!	-
Singh, 2013	Serum calcium	+	-	-	!	!	-

+ Low risk
! Some concerns
- High risk

D1 Randomisation process
D2 Deviations from Intended Interventions
D3 Missing outcome data
D4 Measurement of the outcome
D5 Selection of the reported result

Figure S1. Risk of bias assessment of the included studies investigating the effects of *Cissus quadrangularis* interventions.
