# Supplementary material for: The effects of Cissus quadrangularis on bone-related biomarkers in humans: a systematic review and meta-analysis
Source: BMC Complement Med Ther. 2025 Jul 24;25:286. doi: 10.1186/s12906-025-04995-8 (PMC12288206; doi:10.1186/s12906-025-04995-8)
Supplement: Supplementary file 2 — Supplementary Material 2 [file 12906_2025_4995_MOESM2_ESM.pdf]

Supplementary material 2

Population classified by pathology	Author (year)	Pain	Swelling	Fracture site mobility	Attachment level gain	Bite force	Increment of density	Bone mineral density	Defect resolution	Bone height
		**Clinical Outcome**								
**Bone fracture (n = 5)**										
Bone fracture at various sites (n = 1)	Lingram (2014)	⇩								
Maxillofacial fracture (n = 3)	Singh (2013)	⇩	⇩	⇩	⇔					
	Nayak (2019)	⇔	⇔	⇔		⇔	⇔			
	PG (2023)	⇔		⇔	⇔					
**Total**		4	2	3	2	1	1	0	0	0
**Oral cavity bone diseases (n = 4)**										
Mandibular alveolar ridge distraction (n = 1)	Altaweel (2021)								⇩	⇩
Implant placement (n = 1)	Managutti (2015)	⇩	⇩							
**Total**		1	1	0	0	0	0	0	1	1
**Metabolic bone diseases (n = 1)**										
Postmenopausal women with osteopenia (n = 1)	Benjawan (2022)							⇔		
**Total**		0	0	0	0	0	0	1	0	0
**Total (10)**		5	3	3	2	1	1	1	1	1

⇧ **significant increase**
⇧ **significant decrease**
⇔ **no significant change**

0	1	2	3	4	5

number of studies

Figure S2. Overview of clinical outcome parameters across the included studies
