# Supplementary material for: The effects of Cissus quadrangularis on bone-related biomarkers in humans: a systematic review and meta-analysis
Source: BMC Complement Med Ther. 2025 Jul 24;25:286. doi: 10.1186/s12906-025-04995-8 (PMC12288206; doi:10.1186/s12906-025-04995-8)
Supplement: Supplementary file 3 — Supplementary Material 3 [file 12906_2025_4995_MOESM3_ESM.pdf]

Supplementary material 3

(A) Leave-one-out analysis

a) Serum calcium

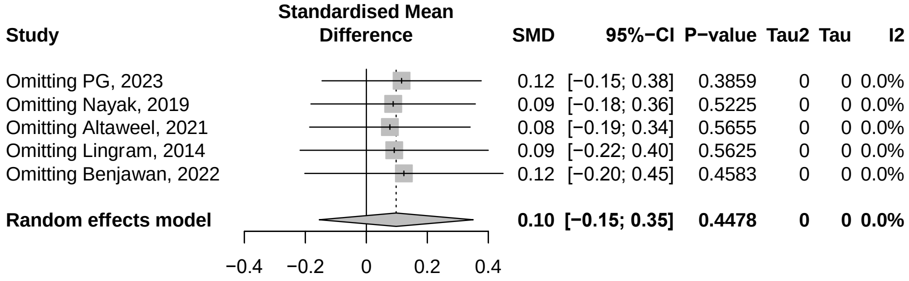

b) Serum ALP

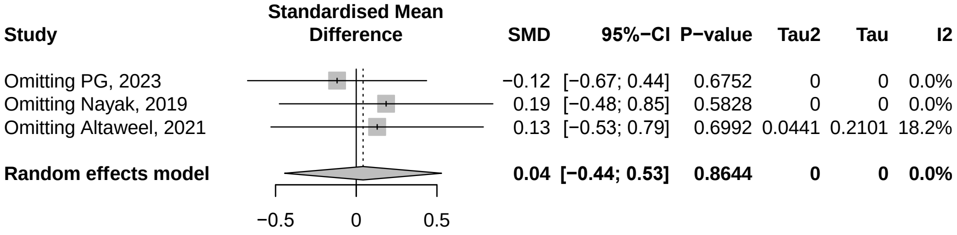

(B) Analysis by common and random effect models

a) Serum PTH

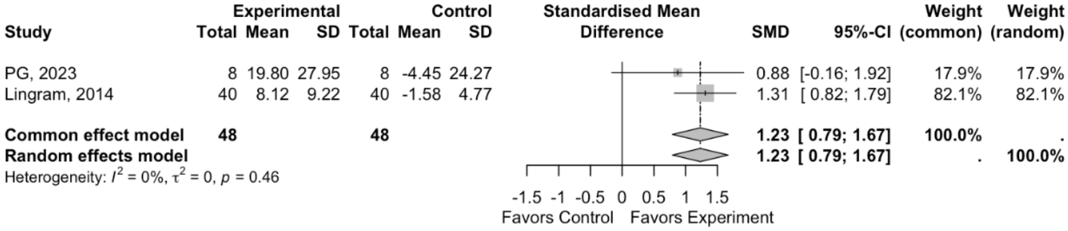

b) Serum Ca

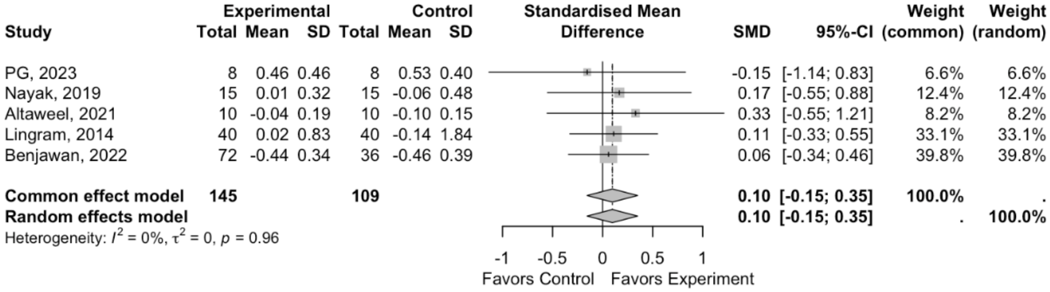

c) Serum P

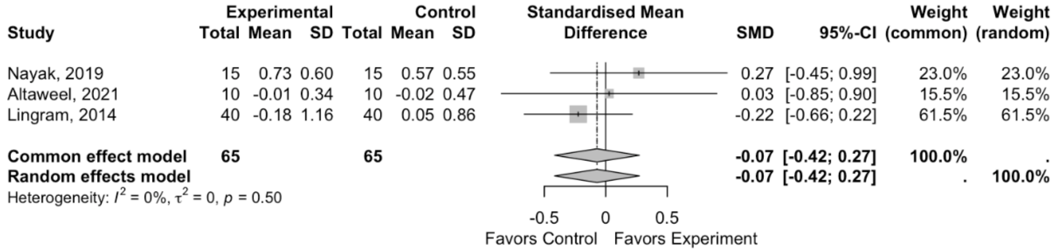

d) Serum ALP

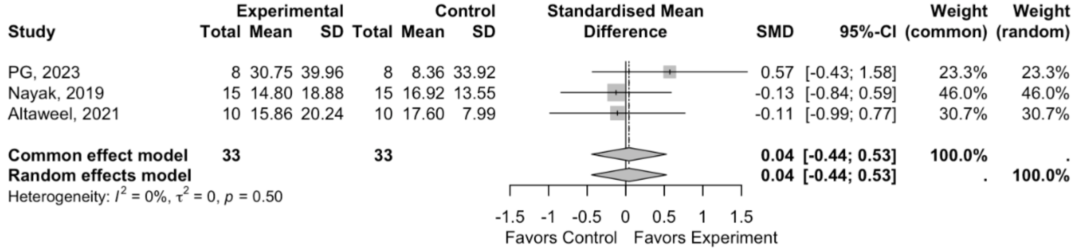

Figure S3 (A) Leave one out analysis of Cissus quadrangularis interventions on bone-related biomarkers: (a) serum calcium and (b) serum alkaline phosphatase. (B) Effect of *Cissus quadrangularis* interventions analyzed by common and random effect models on bone-related biomarkers: (a) serum parathyroid hormone, (b) serum calcium, (c) serum phosphorus, and (d) serum alkaline phosphatase.
